# The Downregulation of *c19orf12* Negatively Affects Neuronal and Musculature Development in Zebrafish Embryos

**DOI:** 10.3389/fcell.2020.596069

**Published:** 2020-12-23

**Authors:** Luca Mignani, Daniela Zizioli, Giuseppe Borsani, Eugenio Monti, Dario Finazzi

**Affiliations:** ^1^Department of Molecular and Translational Medicine, University of Brescia, Brescia, Italy; ^2^Laboratory of Clinical Chemistry, Azienda Socio Sanitaria Territoriale (ASST) Spedali Civili di Brescia, Brescia, Italy

**Keywords:** MPAN, neurodegeneration with brain iron accumulation, zebrafish, neuronal development, *C19orf12* gene

## Abstract

Mitochondrial membrane Protein Associated Neurodegeneration (MPAN) is a rare genetic disorder due to mutations in *C19orf12* gene. In most cases, the disorder is transmitted as an autosomal recessive trait and the main clinical features are progressive spastic para/tetraparesis, dystonia, motor axonal neuropathy, parkinsonisms, psychiatric symptoms, and optic atrophy. Besides iron accumulation in the globus pallidus and substantia nigra, the neuropathology shows features also observed in Parkinson’s Disease brains, such as α-synuclein-positive Lewy bodies and hyperphosphorylated tau. Mutations in the gene have been found in other neurodegenerative disorders, including PD, hereditary spastic paraplegia, pallido-pyramidal syndrome, and amyotrophic lateral sclerosis. The biological function of *C19orf12* gene is poorly defined. In humans, it codes for two protein isoforms: the longer one is present in mitochondria, endoplasmic reticulum, and contact regions between mitochondria and ER. Mutations in the gene appear to be linked to defects in mitochondrial activity, lipid metabolism and autophagy/mitophagy. To increase the available tools for the investigation of MPAN pathogenesis, we generated a new animal model in zebrafish embryos. The zebrafish genome contains four co-orthologs of human *C19orf12*. One of them, located on chromosome 18, is expressed at higher levels at early stages of development. We downregulated its expression by microinjecting embryos with a specific ATG-blocking morpholino, and we analyzed embryonal development. Most embryos showed morphological defects such as unsettled brain morphology, with smaller head and eyes, reduced yolk extension, tilted and thinner tail. The severity of the defects progressively increased and all injected embryos died within 7 days post fertilization. Appropriate controls confirmed the specificity of the observed phenotype. Changes in the expression and distribution of neural markers documented a defective neuronal development, particularly evident in the eyes, the optic tectum, the midbrain-hindbrain boundary; Rohon Beard and dorsal root ganglia neurons were also affected. Phalloidin staining evidenced a significant perturbation of musculature formation that was associated with defective locomotor behavior. These data are consistent with the clinical features of MPAN and support the validity of the model to investigate the pathogenesis of the disease and evaluate molecules with potential therapeutic effect.

## Introduction

The accumulation of iron in specific brain regions, and particularly in the basal ganglia, is a common hallmark of different rare neurological disorders of genetic origin. The term Neurodegeneration with Brain Iron Accumulation (NBIA) was proposed in 2003 to collectively identify patients showing the cerebral sign together with a set of neurological symptoms including, among others, dystonia, spasticity, parkinsonism, neuropsychiatric changes, and optic atrophy or retinal degeneration (Hayflick et al., 2003). Since then, a number of different genetic associations have been identified and now the NBIA disorder category includes at least 12 different types of disease, each linked to sequence variants in specific genes (Levi and Tiranti, 2019) while the idiopathic forms represent about 20% of the cases. The genes coding for ceruloplasmin (CP) and ferritin light chain (FTL) are clearly involved in iron homeostasis and different studies elucidated the mechanisms underpinning the pathology and the accumulation of the metal in the brain parenchyma (Levi and Finazzi, 2014). All the other genes have no evident connection with cellular or systemic iron handling and are involved in different cellular processes, such as coenzyme A biosynthesis, remodeling of membrane lipids, and autophagy (Di Meo et al., 2019).

Together with those in WD repeat domain 45 (*WDR45*), Pantothenate Kinase 2 (*PANK2*), and Phospholipase A2-group VI (*PLA2G6*), mutations in *C19orf12* gene are the most frequent cause of NBIA. Hartig et al. (2011) identified the association in 2011 by conducting a whole exome sequencing on patients of Polish descent. Given the mitochondrial localization of the protein encoded by the orphan gene, the authors proposed to name this NBIA subtype by the acronym MPAN (Mitochondrial membrane Protein Associated Neurodegeneration, OMIM #614298). Since then, several cases carrying different type of mutations in *C19orf12* gene have been reported (Dušek et al., 2018).

Most MPAN cases are transmitted as an autosomal recessive trait, but frameshift mutation in the third exon appear to be associated with a dominant negative effect and have a dominant transmission (Gregory et al., 2019). The main clinical features are progressive spastic para/tetraparesis, dystonia, motor axonal neuropathy, parkinsonisms, psychiatric symptoms, retinal abnormalities, and optic atrophy (Hartig et al., 2011; Horvath et al., 2012). The age of onset is usually within the second decade of life and the clinical progression is slow. Iron accumulation is present in the globus pallidus and substantia nigra, as detected by magnetic resonance imaging, showing T2-weighted hypo-intensity. Neuropathological analysis of patients’ brains demonstrated the presence of axonal spheroids and swellings, α-synuclein-positive Lewy bodies, and hyperphosphorylated tau in the cortex, globus pallidus, substantia nigra, and brain stem (Hartig et al., 2011). Interestingly, mutations in *C19orf12* cause other neurodegenerative disorders such as hereditary spastic paraplegia phenotype 43 (SPG43) (Landouré et al., 2013), pallido-pyramidal syndrome (Kruer et al., 2014), and juvenile Amyotrofic Lateral Syndrome (Deschauer et al., 2012). Investigation of the overlapping features between these disorders may provide important clues about common pathogenic mechanisms.

*C19orf12* maps on human chromosome 19 and encodes for a protein of 141 amino acid residues, which is highly conserved among vertebrates; about 60% of the amino acid residues are identical between the human and zebrafish orthologs. In human, chimpanzee, and chicken, the use of an alternative first exon allows the production of a second and longer isoform of 152 amino acids. It is assumed that the longer form plays a major role in cellular biology and pathology development and most research activity about MPAN focused on it. *In silico* evaluation of the *C19orf12* protein indicated that it contains glycine-zipper motifs, which could be relevant for the interaction with the membrane; the longer isoform is present in mitochondria, endoplasmic reticulum (Landouré et al., 2013), and MAM, contact regions between mitochondria and ER (Venco et al., 2015). No clear information is available about the shorter isoform. *C19orf12* is ubiquitously expressed, but higher levels are found in the brain, blood cells and adipocytes, while transcriptome analysis indicated a co-regulation with genes involved in fatty acid biogenesis and valine, leucine, and isoleucine degradation (Hartig et al., 2011). Even though the function of the protein is still unknown, a putative role in lipid metabolism is suggested (Hartig et al., 2011; Panteghini et al., 2012).

Altogether more than 100 MPAN cases have been reported in the literature (Dušek et al., 2018) up until now. Many mutations hit the putative transmembrane domain and might affect the cellular localization or the mode of interaction with the membranes of the affected protein. Interestingly, a common mutation (p.Thr11Met) hits the eleventh codon of the longer isoform, thus suggesting a more important role for the longer isoform in the pathology. Noteworthy, the mutation also affects the Kozak’s sequence surrounding the starting codon of the shorter isoform, and might affect its expression level. This aspect has not been investigated yet.

The information about the cellular function of *C19orf12* gene is very limited. Venco et al. (2015) overexpressed in HeLa cells the wild-type and two mutant forms (p.Gly58Ser and p.Gln69Pro) of the protein, affecting a residue within a putative glycine-zipper motif in the transmembrane region and a proline in the regulatory domain, respectively. While the first mutant displayed partial changes in cellular localization, both of them affected the response to increased oxidative stress and the induction of the autophagy process observed with the wild-type protein. Altogether, this work suggests a possible involvement of *C19orf12* in controlling autophagy and in particular a role as a sensor of mitochondrial damage and mitophagy. MPAN has been recently modeled in *Drosophila melanogaster* (Iuso et al., 2014), which carries two co-orthologs of the human gene (shorter isoform). The expression of both genes was abrogated by performing RNAi in compound heterozygous mutant strains; this resulted in a phenotype characterized by reduced median lifespan, vacuoles in the brain, and defects in climbing and bang sensitivity.

We have recently exploited the zebrafish animal model to investigate the role of two NBIA genes, namely *pank2* (Zizioli et al., 2015) and *coasy* (Khatri et al., 2016), in embryonal development and gained new insights about the role and relevance of the genes in different tissues and the effects of different types of mutation (Khatri et al., 2018). Here we present the analysis of the *C19orf12* gene in the same experimental system. The zebrafish genome contains four co-orthologs of human *C19orf12*, one on chromosome 18 and the others clustered in tandem on chromosome 7. Available RNA-seq data shows that the gene on chromosome 18 is expressed about 10 times more than the others at early stages of development. We microinjected zebrafish embryos with a morpholino expected to shut off translation of both maternal and zygotic mRNA of this gene. We show that the knockdown of *c19orf12* provides relevant information about the role of the gene during zebrafish development and represents a valuable model to investigate the biological function of the gene and the mechanisms underlining MPAN pathogenesis.

## Materials and Methods

### Zebrafish Maintenance

All the experiments were performed with embryos under the age of 5/7 days post fertilization (dpf), in accordance with the standards defined by the Local Committee for Animal Health (Organismo per il benessere animale, project 211B5-10) and following the Italian and European regulations on animal care. Embryos were generated from AB wild-type and transgenic lines [Tg(*neurog1*:EGFP) Tg(*neurod1*:EGFP)]. Adult fish were housed in 5 L water tanks at 28°C, in a light (14 h) and dark (10 h) timed cycle and fed three meals a day. Each experiment was performed twice or three times, depending on the number of embryos used in each biological replicate. The total number of embryos analyzed is indicated in each figure or figure legend.

### Microinjection and Phenotypic Assessment

Supplementary Table S1 reports the sequences of the morpholinos applied in the experimental procedures. We prepared the injection solution by diluting the morpholino stock solution (1 mM) with 10% phenol red and water to obtain the desired final morpholino concentration. We injected 4 nl of the final solution (0.3 pmol/embryo) into 1–2 cell stage embryos by an Eppendorf FemtoJet Micromanipulator 5171. Embryos were then transferred in fish water containing 0.003% 1-Phenyl-2-thiourea at 28°C to stop the pigmentation process, and development was evaluated at 24 and 48 hpf. Images were captured using Zeiss Axio Zoom V16 equipped with Zeiss Axiocam 503 color digital camera and processed using Zen Pro software from Zeiss.

### Eye and Somite Measurement

We measured the eyes diameter and somite length using the manual graphic length annotation tool available in the Zen Pro software, which automatically calculates the distance between two points. Ten embryos were taken per image to reduce the time between the snapshots avoiding progression of embryos development. We defined the eye diameter as the distance between the anterior surface of the lens and the posterior surface, using jaw, eye centroid and otolith as reference points (Supplementary Figure S1A).

As for somite length, embryos were stained with phalloidin as described. We selected the 10th somite starting from the head, in lateral views of the embryo, and measured the distance between the ventral and the dorsal boundary of the somite, as shown in the Supplementary Figure S1B.

### Whole-Mount *in situ* Hybridization (WISH) Analysis

Embryos at different development stages were collected and fixed using 4% paraformaldehyde in PBS for 2 h at 4°C, dehydrated with an increasing scale of methanol/PBS and transferred in 100% methanol for storage at −20°C. The ORF of the four co-orthologs were amplified (primers in Supplementary Table S1) and cloned in pGem-T-Easy vector (Promega). We synthesized labeled sense and anti-sense probes by T7 and SP6-driven transcription with the digoxigenin labeling kit (ROCHE) according to manufacturer’s instructions. WISH was performed according to a standard protocol (Thisse and Thisse, 2008). Probe hybridization was revealed by overnight incubation with an anti-DIG antibody conjugated with alkaline phosphatase (1:10,000) at 4°C and staining by NBT/BCIP solution. WISH images were captured using a Zeiss Axio Zoom V16 equipped with Zeiss Axiocam 506 color digital camera and processed using Zen Pro software from Zeiss.

### *In vitro* Synthesis of Human Wild-Type and Mutant *C19orf12* mRNAs

The recombinant plasmids (pCMV6-Entry Vector) containing the coding region of human *C19orf12*, both WT and mutant form (c.172G>A) were a generous gift of Dr. Valeria Tiranti (*Fondazione IRCCS Istutito Neurologico Carlo Besta*, Milan, Italy). Linearized plasmids were transcribed with T7 RNA polymerase using the mMESSAGE mMACHINE^TM^ T7 Transcription Kit (Thermo Fisher Scientific, AM1344) according to the manufacturer’s protocol. The product was quantified by My Spect spectrophotometer and used for microinjections (150 pg/embryo).

### Acridine Orange Staining

To analyze the level of cell death, acridine orange staining was performed using a standard protocol (Perkins et al., 2005). Embryos at 48 hpf were dechorionated and incubated for 30 min in acridine orange staining solution (10 mg/l). Embryos were then rinsed three times using fish water, mounted using 80% glycerol and quickly imaged using epifluorescent microscopy (Zeiss Axio Zoom.V16 equipped with Zeiss Axiocam 506 color digital camera and processed using Zen Pro software from Zeiss).

### Lightsheet Imaging

For lightsheet image embryos were first anesthetized using tricaine (0.02%) and included in a low melting agarose matrix 0.5% (Top Vision Low Melting Point Agarose, Thermo Fisher Scientific) then put in the instrument holder. Images were acquired using Zeiss LightSheet microscope V1 supported by ZenPro software. EGFP acquisition was performed using 488–30 nm laser and 505–545 nm filter, phalloidin-TRITC and dsRED with 561–20 nm laser and 585lp filter. Images from the same experiment were taken with the same laser intensity and exposure time to generate comparable images. After the acquisition, 3D images were generated and manipulated using Arivis software. 3D reconstructions of phalloidin fluorescence were manipulated to obtain pictures comparable to each other in terms of fluorescence intensity. Set points for intensity/pixels histogram for the STD and ATG embryos were differently selected to minimize the difference between the signal intensity. 3D reconstructions were exported as a single snap with the same compression setting.

### Birefringence Analysis

Birefringence was quantified by taking images of STD and ATG embryos at 48 hpf under polarized light at the same exposure settings and magnification.

### F-Actin Staining and Somite Size Measurement

Forty-eight hpf embryos were fixed with 4% PFA at 4°C overnight. The embryos were then washed using PBS/0.1% Tween 20 (PBST) three times for 10 min, and permeabilized in 2% TritonX-100 in PBS for 2 h at room temperature. The molecular probe was prepared by diluting phalloidin (Sigma Aldrich) 1:200 in PBS. Embryos were incubated in staining solution for 2 h in the dark at room temperature. Several washes with PBST were performed at 4°C overnight and 2 h at room temperature the following day.

### Quantification of Fluorescence Intensity by ZF-Mapper Application

Pictures of single embryos were taken with the same magnification (40×), laser intensity and exposition using Zeiss Axio Zoom V16 equipped with Zeiss Axiocam 503 color digital camera in 16 bit. Images were converted in 8 bit using Zen Pro software and exported in tiff format. Fluorescence was measured using Z-Mapper software (Yamamoto et al., 2019) with a threshold value of 7; data obtained were represented as the ratio between total pixel intensity and the number of pixels.

### Analysis of Locomotor Behavior

We assessed the spontaneous head-tail coil movement in embryos at 24 hpf. We placed embryos under the Leica MZ16F stereomicroscope (Leica Microsystems Ltd.) and counted the number of movements for 1 min. At least 30 embryos of each type were analyzed. We analyzed the swimming performance of embryos at 48 hpf. A motility wheel, representing four concentric circles at 5, 10, 15, 20 mm diameter, was placed beneath a 60 mm Petri plate containing fish water. Then, we placed a single embryo at the center of the concentric circles. Once the embryo was stable, it was stimulated by softly touching at its tail by the help of poker. Once stimulated, the distance moved by each embryo respective to the concentric circle was recorded. If the embryo failed to cross the first concentric circle, it was re-centered and stimulated again. After five attempts with negative results, it was classified as incapable of crossing the first concentric circle of the motility wheel. We analyzed a total of 40 embryos for each group per experiment.

### Statistical Analysis

Data in the manuscript are a representation of two or more independent experiments with similar results. We performed the statistical analysis using GraphPad Prism 6 software. The significance between various groups was determined by two-way ANOVA, corrected for multiple comparison or by two-tailed unpaired Student’s *t*-test, as indicated in the legend of each figure.

## Results

### *In silico* Analysis

A bioinformatic analysis revealed that *C19orf12* gene is conserved in vertebrates and that four homologous sequences can be found in *Danio rerio*, one on chromosome 18, *zgc:112052*, and three in tandem on chromosome 7 (*si:ch211_260e23.7*, *zgc:101715*, and *si:ch211_260e23.8*). Hereafter, for the sake of simplicity, we will call the genes in the manuscript as follows: (*c19orf12*) *a*, *b1*, *b2*, and *b3*, respectively (Supplementary Table S2).

We analyzed the genomic region surrounding *C19orf12* using Genomicus^1^. The bioinformatic tool showed conserved synteny between the region surrounding human *C19orf12* gene on chromosome 19 and that surrounding *Danio rerio C19orf12* homologs on chromosomes 7 and 18. In a window of 41 genes, human chromosome 19 shows the greatest synteny with *Danio rerio* chromosome 7, with 10 genes other than *b1*, *b2*, *and b3* having conserved positions. *Danio rerio* chromosome 18 shows a lower degree of synteny to human chromosome 19, with only three genes that have preserved their spatial deposition (Supplementary Figure S2A). Similar results were obtained using the Synteny db database^2^.

These data suggest that the four zebrafish genes were the result of the genome-wide duplication event that occurred in teleost fish during evolution, resulting in a gene on chromosome 18 and one on chromosome 7, with a subsequent tandem duplication of the latter to generate two extra copies.

The multiple alignments between the polypeptide sequences of human *C19orf12* and zebrafish co-orthologs show a high degree of identity, except for the first 11 amino acids that are present exclusively at the N-terminal of the human sequence (Supplementary Figure S2B). Using Clustal Omega multiple alignment tool (Sievers et al., 2011), we observed 59.6% identity with the human counterpart for zebrafish protein *a*, 48.2, 51.1, and 55.9% for polypeptides *b1, b2*, and *b3*, respectively (Supplementary Figure S2B and Supplementary Table S3).

A phylogenetic analysis carried out using the human *C19orf12* sequence for the multiple sequence alignment shows that the proteins of the four putative orthologs of *Danio rerio* all segregate in the clade (Supplementary Figure S2C).

All four genes appear to be transcribed, as demonstrated by the presence of EST sequences in dbEST (data not shown) and data from zebrafish transcriptome analysis. RNA-level expression data for the four genes was obtained from a systematic study performed using RNA-Seq reads from nine different developmental stages of the zebrafish embryo (Yang et al., 2013). The RNA-seq data indicate that the four zebrafish genes are transcribed during the development, with no large fluctuations among different developmental stages. Gene *a* appears to be expressed at much higher level than the other three paralogs on chromosome 7 (Supplementary Figure S3). We amplified by RT-PCR the full-length open reading frame of each gene from 100 ng of cDNA from 48 hpf embryos. The quantification of the band intensity shows that gene *a* signal is more than twofold higher than that of the other orthologs (not shown). This semi-quantitative analysis broadly confirms the RNA-seq data described above and indicates that gene *a* is expressed at higher level among the four *C19orf12* orthologs.

### Spatial and Temporal Expression Analysis of Zebrafish *c19orf12* Genes During Embryonic Development

**FIGURE 1:**
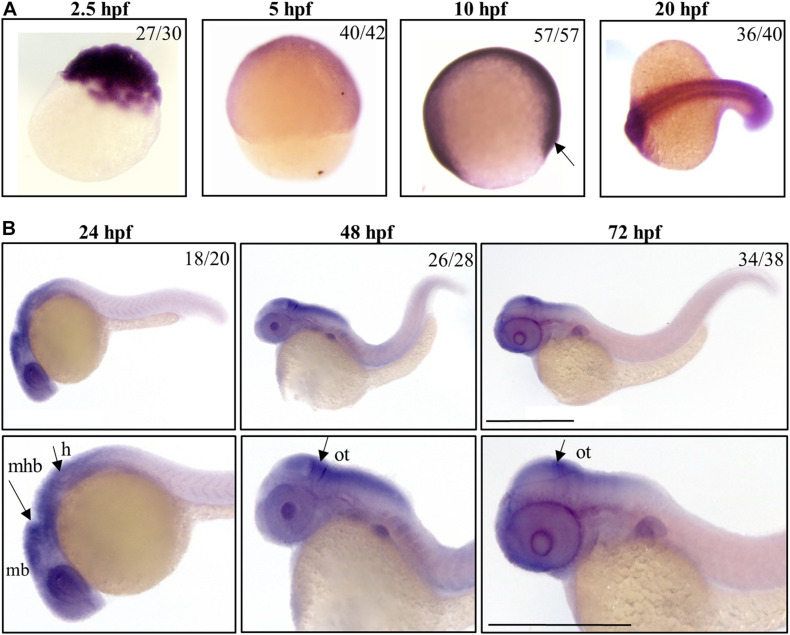
Spatio-temporal analysis of gene *a* expression in zebrafish embryos. Representative images of embryos at early **(A)** or later **(B)** stages of development, hybridized with a probe specific for *c19orf12a* mRNA. Numbers in each panel represent embryos used for the experiment and embryos with the result shown in the image. Each hybridization was performed at least twice. Scale bars = 500 μm. mb, midbrain; mhb, midbrain-hindbrain boundary; h, hindbrain; ot, optic tectum.

We investigated the spatial and temporal expression of zebrafish *c19orf12* genes during embryonic development by WISH at early (from 2.5 till 20 hpf) and later developmental stages (24, 48, and 72 hpf). We used the sense probe for each target gene in parallel control experiments for the analyzed embryonic stages and we did not detect any staining (data not shown), thus confirming the specificity of the staining with the antisense probes. In each experiment, the staining incubation was extended till the appearance of the color was evident and the intensity of the signal provides no quantitative information. A ubiquitous expression of gene *a* is detected at early stages of development, in particular at the cleavage stage (2.5 hpf), thus suggesting its maternal expression (Figure 1). Stable and ubiquitous staining from cleavage to the gastrula stage indicates continuous expression of gene *a* throughout the transition from maternal to zygotic transcription. At 5 hpf gene *a* is clearly expressed in the ectodermal layer. At 10 hpf, a clear signal for gene *a* is present. From 20 hpf, gene *a* appears to be expressed in the somites and the anterior part of the embryo, where the main structures of the central nervous system will develop (Schier and Talbot, 2005). At the described early stages, we could detect a signal for all the orthologs on chromosome 7 (*b1*, *b2*, and *b3* genes), which were broadly expressed in the entire shield (Supplementary Figure S4).

By 24 hpf, the forebrain (most anterior), midbrain and hindbrain (most posterior) structures in the brain are broadly defined and can be easily distinguished by visually identifiable morphogenetics boundaries (Gibbs et al., 2017). At this embryonic stage, gene *a* is clearly expressed in the central nervous system regions, including the midbrain and midbrain-hindbrain boundary. A strong signal is present in the eyes and in the hindbrain, from where originate motor neurons that control the movement of the eyes, jaw, head and body (Vaz et al., 2019). At 48 and 72 hpf, the expression of gene *a* is still well-detectable in tectum, a part of the midbrain where sensory inputs and visual processing take place. These results are in good accordance with those generated by Thisse et al. (2004) and available in the ZFIN expression database.

*b1*, *b2*, and *b3* genes showed a similar expression pattern in CNS structures and partially in the somites at 24 hpf, but the hybridization signals were essentially absent at 48 and 72 hpf (Supplementary Figure S5).

Taken together, these data indicate that, starting from early stages of development, *c19orf12* genes are expressed in the developing embryos; at 20 hpf they shared a similar expression pattern in central nervous system structures and somites but at 24 hpf, there is a different expression of the four orthologs in CNS. Actually, the mRNA of gene *a* is clearly detected in the main structures of the developing brain while transcripts of the other three orthologs are not so clearly found in the same brain structures.

### Morphological Analysis of Embryos Injected by *c19orf12a*-Specific Morpholinos

Based on the results from the WISH study and the available RNA-seq data, we focused our attention on gene *a* and designed a splice-inhibiting morpholino (SI-MO, Supplementary Table S1) to knock down its expression at early stages of development. The SI-MO was directed against the exon 2-intron 2 junction site of gene *a* mRNA and predicted to block the splicing of the immature mRNA by inducing the retention of intron 2. In parallel, we used a standard morpholino (STD-MO, Gene Tools Inc., Supplementary Table S1) without any specific target in zebrafish as a negative control.

We injected SI-MO into 1–2 cell stage embryos at different doses, and the effect on the embryonic development was observed at 24 and 48 hpf (Supplementary Figure S6). Even at the highest dose of 1 pmol/embryo, we did not observe any evident morphological changes and SI-MO-injected embryos appeared similar to the non-injected ones. Amplification of the target mRNA resulted in bands of similar intensity in both control and SI-MO-injected embryos (Supplementary Figure S6C), hence we concluded that the SI-MO was ineffective at reducing the expression of gene *a* in zebrafish embryos. We checked for changes in the genomic sequence targeted by the morpholino in our fish strains as the possible cause of this phenomenon and we found none.

We then switched to a translation-inhibiting morpholino (ATG-MO) complementary to the −17/+5 region of gene *a* mRNA. We set up a dose response assay to investigate its efficacy. Clear morphological abnormalities associated with increased lethality were evident for all the injected doses (0.3, 0.5, 0.75, 1.0 pmol/embryo) (Supplementary Figure S7). We selected the lower dose of 0.3 pmol (2.5 ng)/embryo, which was associated with the lowest mortality, for all further studies.

**FIGURE 2:**
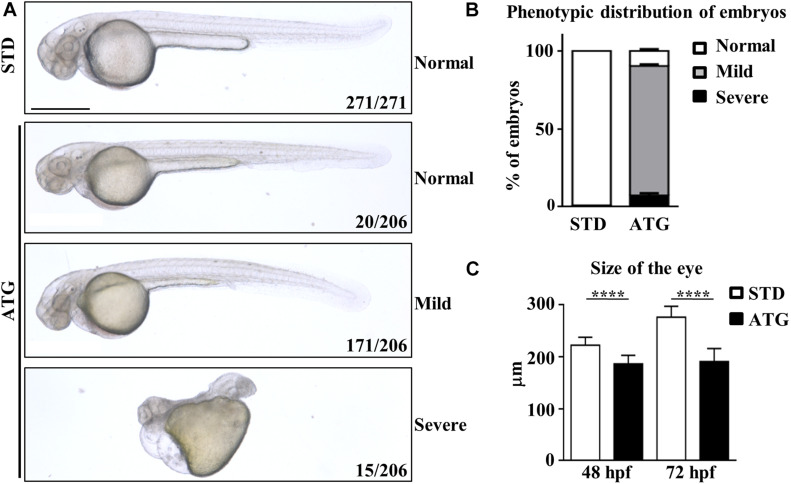
Morphological analysis of embryos micro-injected with an ATG-blocking morpholino. Embryos were injected with the gene *a*-specific (ATG) or the control (STD) morpholino at 1/2 cell stage and analyzed at the microscope at 48 and 72 hpf. **(A)** Representative images showing the main features of controls and morphants at 48 hpf. Numbers in each panel represent total embryos used for the experiment and embryos with the phenotype shown in the image. **(B)** The graph shows the percentage distribution of embryos with normal or abnormal (mild and severe) morphology in each microinjection category. **(C)** The graph show the mean size of the eye diameter in controls and morphants (*N* > 40) at 48 and 72 hpf. Size bar = 500 μm. ****P < 0.0001 (unpaired, two-tailed *T*-Test). Experiments were repeated at least three times.

We categorized the phenotype induced by the morpholino according to the severity of the morphological features of the embryos (Figures 2A,B). The large majority of them (171/206, 83.0%) showed perturbed brain morphology with smaller brain size and eyes, reduced yolk extension along with a curved and thinner tail, and defected somite development. They were included in the mild category. A small number of embryos (15/206, 7.3%) were characterized by a dramatic alteration of the structure, with severe perturbation of the antero-posterior axis, cardiac edema and compromised somite development; they were included in the severe category. The remaining embryos (20/206) showed a normal phenotype. The embryos with mild phenotype were used for all further experiments. ATG-MO-injected embryos showing a mild phenotype were analyzed for the diameter of the eye at 48 hpf. The STD-MO-injected embryos were used as a reference. The eye diameter was found to be significantly reduced in ATG-morphants (186.3 ± 16.4 μm) compared to not-injected embryos (NI) and STD-morphants (250.4 ± 18.03 and 222.4 ± 15.2 μm, respectively, Figure 2C).

The abnormal morphological features persisted for a longer time and worsened in the large majority of embryos (>90%), with an aggravation of the curvature of the tail (not shown). All the morphants died between 5 and 7 dpf.

### Analysis of the Specificity of the Morphological Phenotype

**FIGURE 3:**
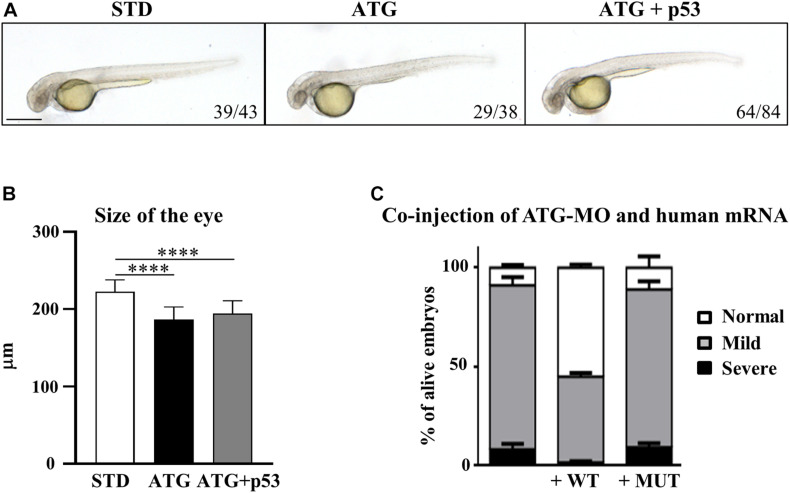
Assessment of the specificity of the phenotype. **(A)** Embryos were injected with ATG-MO for gene *a* alone or together with an equimolar dose of p53-MO. **(B)** The graph shows the size of the eyes in embryos as in **(A)**. Two biological replicates were performed. ****P < 0.001. **(C)** Embryos were injected with ATG-MO for gene *a* alone or together with human *C19orf12* mRNA, either WT or MUT, and the morphology analyzed at 48 hpf. The graph shows the percentage distribution of embryos in the different phenotypes (normal, mild, and severe) in the three categories of embryos. At least three experiments were performed and more than 150 embryos of each type were analyzed.

Even though the staining of ATG-MO-morphants with acridine orange did not show an increase of cell death at 48 hpf (Supplementary Figure S8), we checked for possible *p53*-dependent off-target effects (Robu et al., 2007) by co-injection of equimolar doses of ATG-MO and a morpholino targeting *p53* expression (Gene Tools Inc., Supplementary Table S1). The downregulation of *p53* did not affect the nature and the percent distribution of the morphological abnormalities associated with the blockage of gene *a* translation, including the presence of smaller eyes (Figures 3A,B). Even though we observed a partial recovery of the yolk extension morphology, the result allows to exclude major ATG-MO off-targeting effects.

To validate the cause-effect relationship between the reduced expression of *c19orf12a* gene and the morphological defects observed in ATG-morphants, we evaluated the ability of the synthetic human *C19orf12* mRNA to rescue the abnormal phenotype. For this purpose, we synthesized *in vitro* human wild-type (WT) and mutant (MUT) mRNA, carrying the mutation c.172G > A (G58S). To evaluate possible toxic effect induced by the microinjection of human *C19orf12* mRNA, we injected 150 pg/embryo of either mRNAs and monitored their development until 48 hpf. No changes in survival or morphology were evident in mRNA-injected embryos when compared to mock-injected ones (not shown). Then, we co-injected the same dose of each synthetic mRNA with ATG-MO (0.3 pmol/embryo). The embryos co-injected with ATG-MO and WT-mRNA (82%, *n* = 305/371) or ATG-MO and MUT-mRNA (78%, *n* = 127/163) showed no significant change in the survival rate when compared to the embryos injected with ATG-MO alone (74%, *n* = 290/392) (Supplementary Figure S9).

Nonetheless the expression of the human WT mRNA significantly increased the number of embryos showing a normal phenotype, which were about 55% of the alive embryos (167/305) vs. 8.3% (*n* = 24/290) observed in ATG-MO-injected embryos. About 43% (*n* = 132/305) of the co-injected embryos still showed a mild phenotype, but less than 2% (*n* = 6/305) a severe one (Figure 3C).

On the contrary, the type and percentage distribution of the morphological features observed upon the co-injection of ATG-MO and MUT-mRNA were similar to those observed when ATG-MO alone was injected (Figure 3C). Normal embryos represented 9.4% (14/127) of the survived embryos, while embryos with mild or severe abnormalities were 79.5% (101/127) and 11% (12/127), respectively. The differences in the percentage of embryos with normal and mild phenotype observed between ATG-MO-WT-mRNA co-injected embryos, and ATG-MO-injected embryos was statistically significant (*p* < 0.001). This suggests that the WT human mRNA was able to overcome the deficiency of *c19orf12a* mRNA, by significantly increasing the number of embryos with normal phenotypic development as compared to that observed in the ATG-MO-injected group. On the contrary, the injection of the MUT-mRNA did not affect the aberrant development. This result further confirms the specific relationship between the knock-down of gene *a* mRNA and the perturbed development observed in ATG-morphants.

### Analysis of Neuronal Development

We showed that *C19orf12a* is highly expressed in the central nervous system during early developmental stages. Its downregulation induces morphological anomalies in the head. To gain insight into the involvement of the gene in zebrafish neurogenesis we took advantage of transgenic lines expressing EGFP under *neurogenin1* (*neurog1*) or *neuronal differentiation 1* (*neurod1*) promoters, two transcription factors known to play important roles in neuronal development.

**FIGURE 4:**
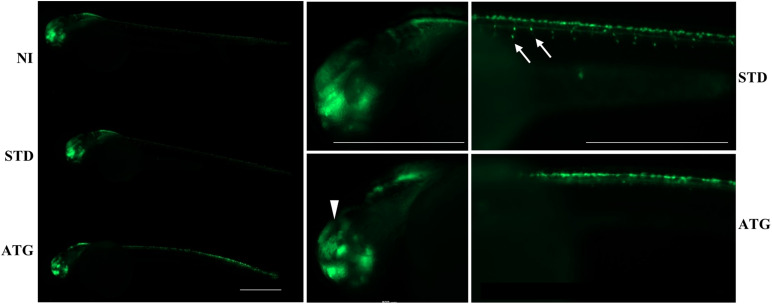
*neurog1*-dependent EGFP fluorescence in embryos injected with control (STD) or ATG morpholinos. Lateral views of Tg(*neurog1*:EGFP) embryos either injected or not with STD or ATG morpholinos, at 48 hpf, with different magnifications. The arrowhead points to the region in the midbrain (tectum opticum) that shows a clear reduction of the signal in morphants. Arrows point to drg neurons, normally labeled in STD-injected embryos, but not visible in morphants. Size bar = 500 μm. Experiments were repeated at least three times and altogether more than 120 embryos of each type were viewed.

**FIGURE 5:**
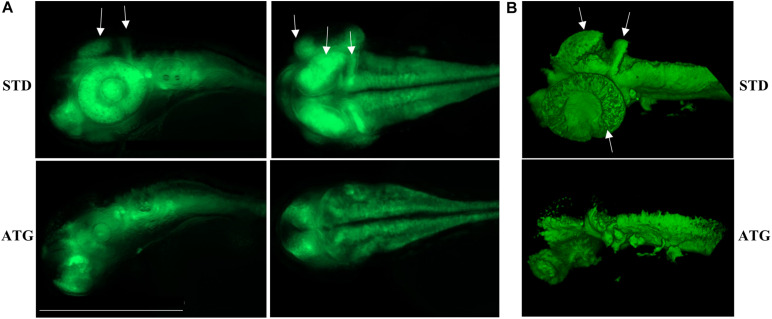
*neurod1*-dependent EGFP fluorescence in embryos injected with control (STD) or ATG morpholinos. **(A)** Representative lateral and dorsal views of Tg(*neurod1*:EGFP) embryos either injected with STD or ATG morpholinos, at 48 hpf, Size bar = 500 μm. Experiments were repeated at least three times and altogether more than 150 embryos of each type were analyzed. **(B)** Lightsheet imaging of embryos described in panel **(A)**. Four embryos with mild morphological phenotype were randomly selected and analyzed in two different experiments. Arrows point to the regions that are clearly missing or less visible in ATG-morphants.

We microinjected Tg(*neurog1*:EGFP) embryos with 0.3 pmol of STD-Mo or ATG-MO and evaluated the transgene expression at 48 hpf. STD-MO-injected embryos showed no difference in comparison with not-injected ones (Figure 4) *neurog1*-driven fluorescence was evident in CNS regions corresponding to the epiphysis (e), the telencephalon (t), the dorsal midbrain (m), the hindbrain (h), Rohon Beard sensory neurons (rb), and dorsal root ganglia neurons (drg) were positive for EGFP expression in the trunk. In general, ATG-MO-injected embryos had a similar pattern of EGFP expression, but specific brain regions or cells were not decorated. Fluorescence was clearly missing in the midbrain, in particular at the level of the optic tectum. In the trunk, drg neurons were not labeled and rb fluorescence intensity appeared to be increased or abnormally distributed (Figure 4). These features were confirmed by lightsheet analysis; the reconstruction of the 3D distribution of EGFP fluorescence in 48 hpf embryos clearly evidenced the lack of EGFP signal in the rostral part of the CNS and in drg neurons (Supplementary Figure S10). This indirect analysis of *neurog1* expression suggests that downregulation of gene *a* affects normal neuronal development of specific brain areas and neuron types. To further investigate this aspect we repeated the same procedure using Tg(*neurod1*:EGFP) embryos and performed fluorescent imaging at 48 hpf. The comparison between STD-MO and ATG-MO-injected embryos revealed significant changes in EGFP fluorescence: the signal was missing or strongly reduced in the optic tectum and the midbrain-hindbrain boundary. Fluorescence in the retina was also dramatically reduced (Figure 5A). The changes in the *neurod1*-dependent signal were confirmed at the lightsheet imaging (Figure 5B).

Altogether, the analysis of the transgenic lines suggests that normal expression of gene *a* at early stages of development is required for determination and/or differentiation of specific neuronal populations such as the drg neurons in the trunk or the cells located in the retina, the tectum opticum and the midbrain-hindbrain boundary.

### Analysis of Musculature Development

**FIGURE 6:**
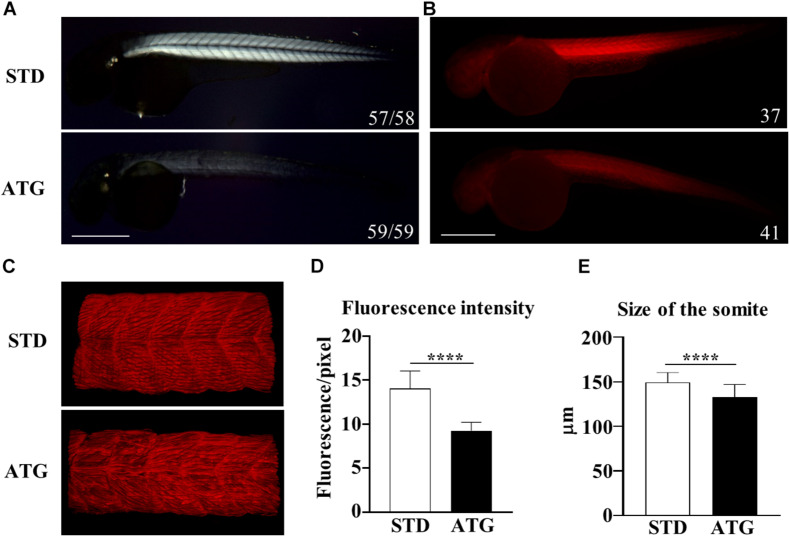
Birefringence analysis and F-actin staining. **(A)** Birefringence quantification in embryos injected with STD or ATG morpholino at 48 hpf. **(B)** Embryos were incubated with fluorescent phalloidin to stain F-actin. Representative lateral views of stained embryos showing the clear reduction of the total fluorescence intensity in ATG-morphants. Images were acquired with the same parameter, as described in section “Materials and Methods.” **(C)** Lightsheet imaging of a section of the trunk in the embryos as in **(A)**. The image of the ATG-morphant was adjusted in order to show a fluorescence intensity comparable to that of the control embryo (STD). **(D)** Quantification of the fluorescence of embryos shown in **(A)**, acquired by ZF-Mapper software. **(E)** Mean size of the trunk in control and ATG-morphants at 48 hpf, measured as described in section “Materials and Methods.” Three (birefringence) and two biological replicates (phalloidin staining) were performed. Numbers in each image indicate the total number of embryos analyzed. Size bar = 500 μm. ****P < 0.0001 (unpaired, two-tailed *T*-test).

The WISH analysis revealed expression of *c19orf12a* also in the myotome. Embryos injected with the ATG-MO (mild phenotype) presented with a curved trunk/tail associated with a reduced dorsal-ventral axis size (Figure 2A), but the overall structure appeared to be normal. To analyze in deeper detail the myotome structure, we assessed the birefringence pattern under polarized light in embryos at 48 hpf (Smith et al., 2013). Control embryos showed bright birefringence, indicative of a highly organized skeletal muscle. On the contrary, ATG-morphants showed an overall reduction in birefringence intensity, suggestive of a disorganized skeletal muscle structure (Figure 6A). Then, we stained STD- and ATG-MO-injected embryos at 48 hpf with fluorescent-conjugated phalloidin that allows for visualization of filamentous F-actin. The comparison with control embryos clearly revealed a strong reduction of fluorescence intensity in MO-injected embryos. The quantification of the signal by ZF–Mapper freeware analysis documented a decrease of about 35% of the fluorescent signal (Figures 6B,D). When the structure of F-actin filaments in the muscles was observed by lightsheet imaging, we could detect a perturbation of the regular, parallel and V-shaped distribution of fibrils within each muscle segment (Figure 6C). The mean size of the trunk was also reduced in ATG-morphants (Figure 6E). The results let infer a connection between *c19orf12* function and normal myotome development.

### Assessment of the Locomotor Behavior

Embryos injected with the *c19orf12a*-specific morpholino showed impaired development of specific CNS structures and musculature. To assess possible functional consequences of these perturbations, we set up the analysis of the locomotor activity of embryos. The first movement that can be detected from 17 hpf is the slow and alternating flipping of the tail. Rapid tail coils can be evoked by touch stimuli from 21 hpf, and finally spontaneous swimming can be observed from 27 hpf (McKeown et al., 2009).

**FIGURE 7:**
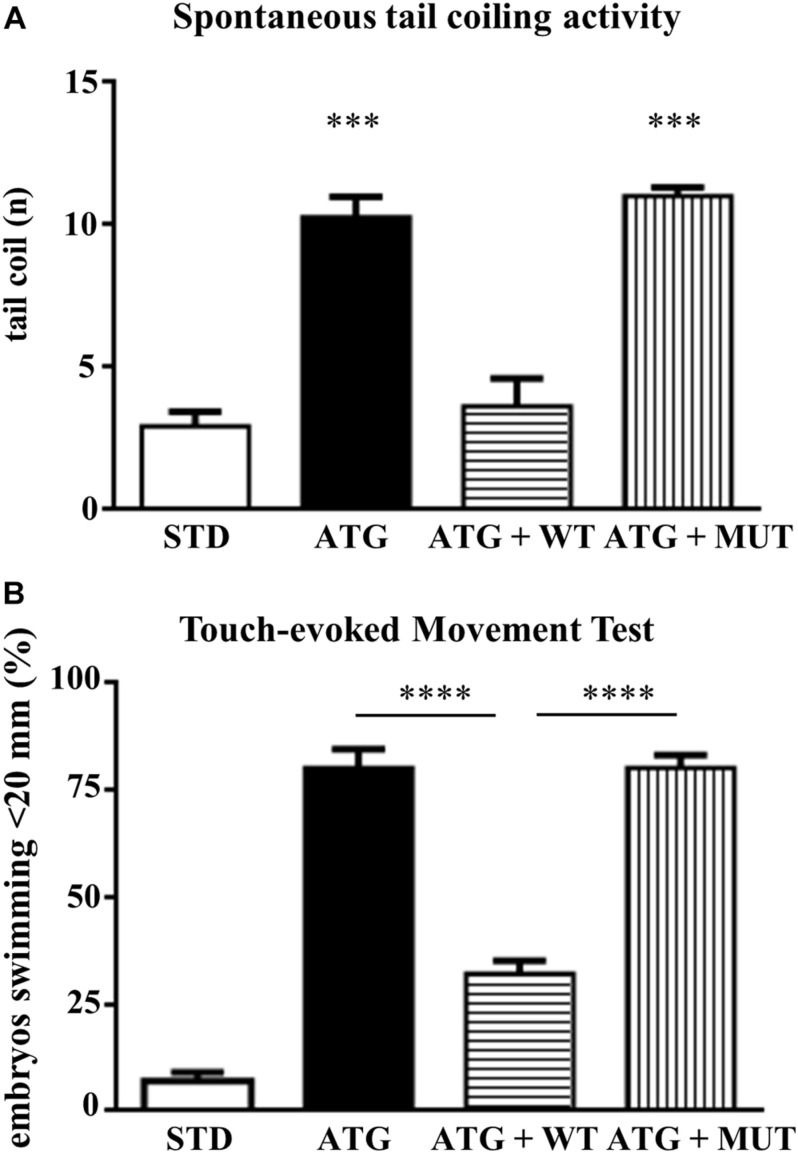
Analysis of locomotor behavior. **(A)** The graph shows the number of spontaneous flipping movements during 1 min observation in embryos at 24 hpf, injected with STD or ATG morpholino, together with either WT or MUT human *C19orf12* mRNA. **(B)** The graph resumes the main results from the analysis of the swimming performance of embryos of the same categories as in **(A)**, at 48 hpf in the touch-evoked test. Bars represent the percentage of embryos covering a distance lower than 20 mm. ****P < 0.0001 (One way ANOVA, corrected with Tukey’s multiple comparisons test).

We investigated the effects of knocking down *c19orf12a* expression on locomotor activity by comparing first the spontaneous coiling contractions at 24 hpf, and then the touch-evoked swimming at 48 hpf. Embryos at 24 hpf move 3–5 times/min (Basnet et al., 2017). We counted the number of spontaneous movements of each injected and control embryo for 1 min. The number of spontaneous tail coil movements was significantly increased at 24 hpf in embryos injected with ATG-MO (mean = 10.35 movements/embryo/min). Interestingly, the co-injection of WT-mRNA prevented the increase in the number of head-tail flipping movements (mean = 4.79), whereas the co-injection of MUT-mRNA failed in rescuing the abnormal behavior (mean = 12.37, Figure 7A).

Then, we performed a touch-evoked movement test according to procedure previously described (Khatri et al., 2018). Almost all (95%) control embryos were able to swim more than 20 mm (Figure 7B). The opposite was observed for embryos injected with ATG-MO; even though we selected embryos with mild phenotype, only a few of them (20%) swam more than 20 mm and about 70% covered less than 10 mm (Supplementary Figure S8) and all required multiple stimuli. The co-injection of ATG-MO and human WT-mRNA appeared to ameliorate the motor performance of morphants, since more than 67% of them were able to swim for the longest distance (Figure 7B). The embryos co-injected with MUT-mRNAs showed similar results as ATG-MO injected embryo (38%, 22%, 20%, 20%; <5, <10, <20, and >20 mm, respectively) (Supplementary Figure S11).

This analysis clearly shows that the downregulation of gene *a* expression is associated with a significant loss of motor performance of embryos at 48 hpf.

## Discussion

Even though the genetic association between *C19orf12* gene and MPAN was established almost 10 years ago (Hartig et al., 2011), the biological function of the gene and the processes involved in disease development are poorly defined and a single *Drosophila* model is available for *in vivo* studies. Therefore, no therapeutic strategy is available yet. In an attempt to fill this gap, we decided to exploit zebrafish embryos to model this rare disorder. Several features of zebrafish embryos together with the large number of experimental tools to investigate them concur to the choice of this animal model as a valuable system to dissect disease mechanisms involved in neurodegeneration and to screen for possible therapeutic targets (Martín-Jiménez et al., 2015). Four different *c19orf12* co-orthologs are present in zebrafish. According to available RNA-seq data, the gene on chromosome 18, that we named gene *a*, is expressed at much higher level than the ones on chromosome 7. Since our WISH and RT-PCR analysis essentially confirmed this piece of information, we concentrated our study on gene *a*. While it is ubiquitously expressed in the early stages of development, it becomes restricted to the CNS from 24 hpf on. The eyes, the optic tectum, the hindbrain and the midbrain-hindbrain boundary are the regions more consistently decorated by the antisense probe for gene *a*. This suggests an involvement in the development of selected brain areas. There is no precise information about the tissue and brain distribution of human *C19orf12* expression. Iron accumulates in the globus pallidus and in the substantia nigra, but neuropathology, and particularly Lewy bodies, is more widespread in the brain of patients, at least at late stages of the disease (Hartig et al., 2011; Hogarth et al., 2013). Of interest, many MPAN cases present with optic atrophy. The intense hybridization signal in the eyes of embryos, together with the staining in the optic tectum, where retina projections of the retinal ganglion cells terminate, seem to indicate a functional conservation for *c19orf12* gene in human and zebrafish.

We successfully knock down gene *a* by the injection of a specific ATG-morpholino and obtained a range of embryonic and larval phenotypes potentially relevant to MPAN disease. Due to the absence of an antibody capable of recognizing zebrafish *C19orf12* protein, we could not document the blockage of mRNA translation. Nonetheless, we could demonstrate the robustness of the observed phenotype by complementary approaches. First, the co-injection of a *p53*-specific morpholino allowed us to exclude off-targeting effects associated with the induction of *p53* activity. Second, the phenotypic rescue obtained by the co-injection of wild-type human *C19orf12* mRNA (and not of the mutant form) proved the functional connection between zebrafish and human *c19orf12* genes and confirmed the specific association between the knock down of gene *a* and the documented embryonic phenotype. Noteworthy, the rescue observed in our experiments was not complete. This could be due to technical aspect such as the stability of the exogenous mRNA *in vivo* or, alternatively, it could suggest an incomplete functional overlap between the longer form of the human protein and the shorter zebrafish one.

The microscopic observation of morphants revealed a smaller head with poorly defined brain structures and reduced eye size, curved and thinner tail, reduced yolk extension. This is in line with the WISH results that highlight the specific expression of gene *a* in the brain and in somites, suggesting a role for the gene in neural and muscle development. Interestingly, a similar aberrant morphology is found in zebrafish experimental models of other human neurodegenerative disorders with early onset (Mahmood et al., 2013; Schaffer et al., 2014) and may be an indication of common biological processes. A closer look at neuronal development was obtained with transgenic lines expressing EGFP under the control of *neurog1* or *neurod1* promoter, which are a powerful tool for the study of neural circuits and brain structures (Satou et al., 2013). *Neurog1* is a basic Helix-Loop-Helix (bHLH) transcription factor involved in determination of neuronal precursor cells in lower vertebrates (Lee, 1997). In zebrafish, its expression starts in the neural plate at the end of gastrulation and progressively expands to larger domains. At 24 hpf, it is detected in the posterior midbrain, the optic chiasma, the ventral and dorsal diencephalon (Korzh et al., 1998). At 48 hpf it is expressed in the epiphysis, the telencephalon, the dorsal midbrain, the hindbrain, in Rohon Beard sensory neurons and in dorsal root ganglia neurons (Blader et al., 2003).

Zebrafish *neurod1* codes for a bHLH factor promoting neuronal differentiation shortly after determination by *neurog1* (Blader et al., 1997). Its expression starts later than that of *neurog1* and partially overlaps with it. At 24 hpf, *neurod1* transcript is particularly evident in the olfactory bulbs, pineal gland, inner ear, midbrain, hindbrain, and neural tube. It is not detected in the developing retina till 31 hpf, when the dorsal-to-ventral wave of neurogenesis starts. By 48 hpf, both the inner and the outer retina present *neurod1*-positive cells (Mueller and Wullimann, 2002, 2003; Thomas et al., 2012). This approach confirmed the significant involvement of the retina and the optic tectum. The midbrain-hindbrain boundary, the hindbrain and dorsal root ganglia were also missing or with reduced fluorescence. Surprisingly, we did not detect an increase in cell death by the acridine orange staining at 48 hpf. To reduce the chance of off-target effects, we concentrated our analysis on embryos exposed to low doses of morpholino and with the mildest morphological phenotype. Even at the lowest dose of ATG-MO, we detected a sharp increase in the number of dead embryos at 48 hpf (from about 5% in control embryos to more than 20% in morphants). When combined with the embryos showing a severe phenotype, about 30% of ATG-MO-injected embryos are essentially not viable at 48 hpf. We can speculate that the strongest reduction of gene *a* expression is not compatible with life. The defects in surviving embryos could be linked to blockage of cell proliferation or delayed differentiation. Some *neurog1*-positive drg neurons appear at later stages of development (72 hpf), but they are reduced in number and do not show axonal projections; at the same time, most of the phenotypic abnormalities, including the reduced eye size and the defective expression of neuronal markers persist in surviving embryos. This let us infer that the reduction of *c19orf12* expression irreversibly affects specific and early developmental pathways that do not recover with the progressive degradation of the ATG-morpholino.

The defects in neuronal development were associated with locomotor behavior abnormalities, i.e., an increased frequency of spontaneous tail coiling movements at 24 hpf and a reduced gliding response in the touch evoked test at 48 hpf. The spontaneous contractions of the trunk originate in the spinal cord and essentially depend on primary motoneurons innervation (Brustein et al., 2003). Changes in the frequency of tail coil activity may depend on defected early neurogenesis, as shown by the neurotoxic effects of chemicals including cadmium, fluoxetine, and citalopram (Zindler et al., 2019). Since periods of quiescence between tail coils are controlled by glycinergic signaling (Knogler et al., 2014), it is possible to speculate a specific sensibility of glycinergic neurons to *c19orf12* defects. WISH for glycine transporters revealed the presence of this type of neurons in the rostral spinal cord at 20 hpf (GlyT2 and GlyT1) and in the hindbrain and midbrain at 24 and 48 hpf (GlyT1) (Chalphin and Saha, 2010); thus a co-localization with gene *a* is possible.

The touch evoked swimming requires more mature neuronal circuits and involves the activation of hindbrain reticulospinal neurons and spinal cord interneurons by stimuli provided by rb and drg neurons (Brustein et al., 2003). The analysis of *neurog1*-driven expression of EGFP in zebrafish embryos at 48 hpf revealed an almost complete absence of drg sensory neurons together with irregular distribution of rb neurons. A defect in sensing the mechanical stimulus could justify the poor motor performance of gene *a* morphants. The requirement for repetitive stimuli to elicit the escape response further supports this interpretation. At the same time, given the brain distribution of *c19orf12a*, we cannot exclude the involvement of hindbrain motor neurons.

Altogether, we think that zebrafish embryos injected with the ATG-MO for *c19orf12a* gene represent a valuable model for the autosomal recessive forms of MPAN disorder. They provided relevant information about CNS regions and neuronal types affected by gene *a* loss-of-function, an essential starting point to investigate the involved molecular mechanisms. At the same time, the morphological and functional features shown by morphants represent an important starting point to test molecules for potential therapeutic strategies. We are considering the possibility to generate fish carrying gene *a* mutations exerting a dominant-negative effect. They would represent an important model to further explore *c19orf12* functioning and MPAN development.

## Data Availability Statement

The raw data supporting the conclusions of this article will be made available by the authors, without undue reservation, to any qualified researcher.

## Ethics Statement

Ethical review and approval was not required for the animal study because the study exclusively involved the use of zebrafish embryos and therefore did not require the approval of an animal ethics committee. Nonetheless, the study was performed in accordance with the standards defined by the Local Committee for Animal Health (Organismo per il benessere animale) and following the Italian and European regulations on animal care.

## Author Contributions

LM and DZ performed the experiments. LM, DZ, and DF conceived the experiments and evaluated the results. LM, DZ, GB, and DF prepared the figures. EM corrected the manuscript. All authors contributed to the article and approved the submitted version.

## Conflict of Interest

The authors declare that the research was conducted in the absence of any commercial or financial relationships that could be construed as a potential conflict of interest.

## Footnotes

1 https://www.genomicus.biologie.ens.fr/genomicus-100.01/cgi-bin/search.pl

2 http://syntenydb.uoregon.edu/synteny_db/
