# Supplementary material for: The Downregulation of c19orf12 Negatively Affects Neuronal and Musculature Development in Zebrafish Embryos
Source: Front Cell Dev Biol. 2020 Dec 23;8:596069. doi: 10.3389/fcell.2020.596069 (PMC7785858; doi:10.3389/fcell.2020.596069)
Supplement: Supplementary file 1 [file Data_Sheet_1.pdf]

(A) **(B)**

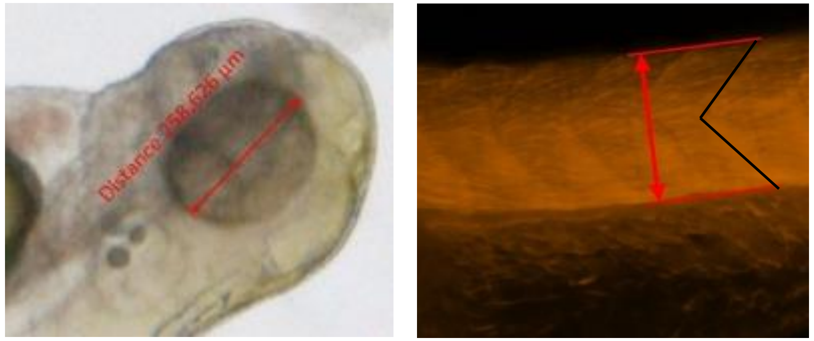

Figure S1. Measurement of eyes and somite size.

We measured the eyes diameter (A) and somite length (B) using the manual graphic length annotation tool available in the Zen Pro software. The eye diameter correspond to the distance between the anterior and posterior surface of the lens surface, using jaw, eye centroid and otolith as reference points. As for somite length, we measured the distance between the ventral and the dorsal boundary of the 10th somite, in lateral views of embryos stained with fluorescent phalloidin.

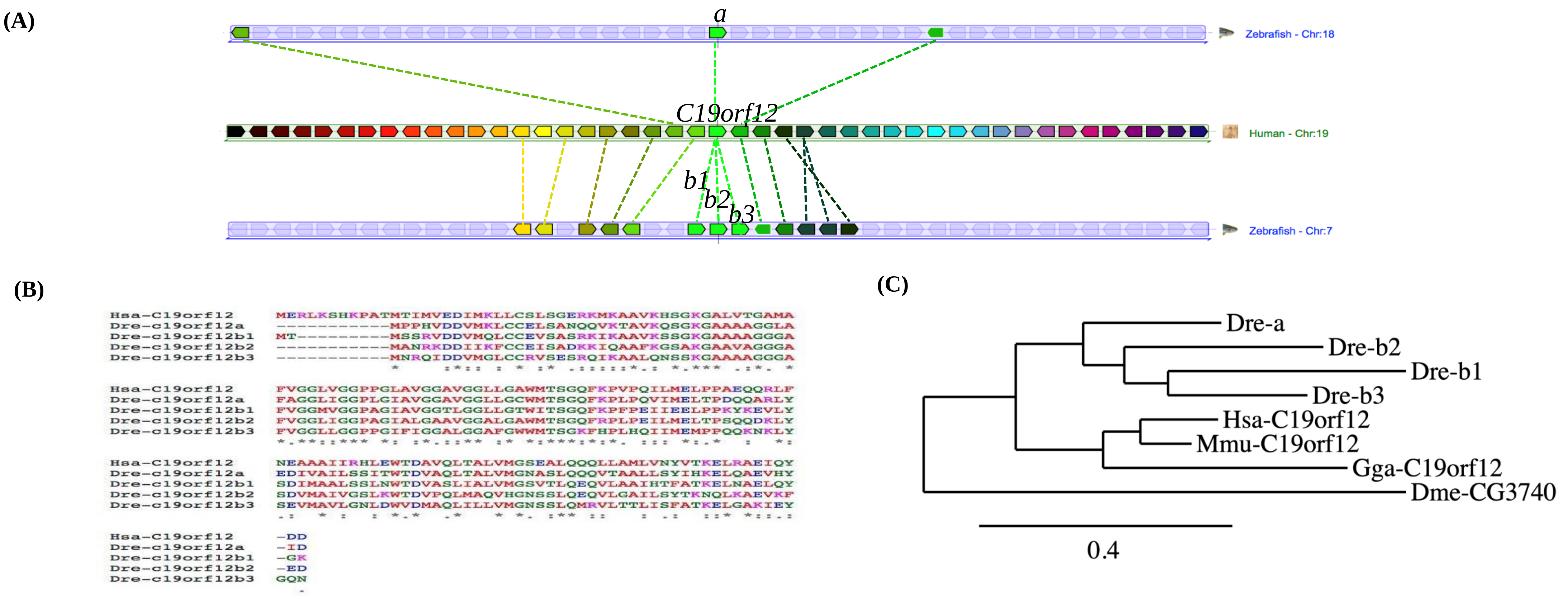

Figure S2. In silico analysis

(A) Graphical representation of conserved synteny around the *C19orf12* locus between *Homo sapiens* chromosome 19 and *Danio rerio* chromosomes 7 and 18, generated using the Genomicus synteny browser. Dashed lines connect orthologous gene pairs within the clusters. (B) Multiple sequence alignment of human (Hsa) C19Orf12 protein (NP_001026896) and zebrafish (Dre) a (NP_001017665), b1 (XP_021333620), b2 (XP_005166511), and b3 (XP_021333621) polypeptides performed with Clustal Omega at EMBL-EBI. Residues are colored according to their physicochemical properties. Asterisks (*) indicate positions which have a single, fully conserved residue, colons (:) indicate conservation between groups of strongly similar properties while periods (.) indicate conservation between groups of weakly similar properties. (C) Unrooted tree showing phylogenetic analysis results for human (Hsa), mouse (Mmu, NP_082442), chicken (Gga, NP_001264595), Drosophila (Dme, NP_569932) and zebrafish (Dre) C19orf12 polypeptides generated using the Phylogeny.fr web service. A square bracket evidences the four zebrafish C19orf12-related proteins. Branch support values are displayed. The horizontal bar represents a distance of 0.4 substitutions per site.

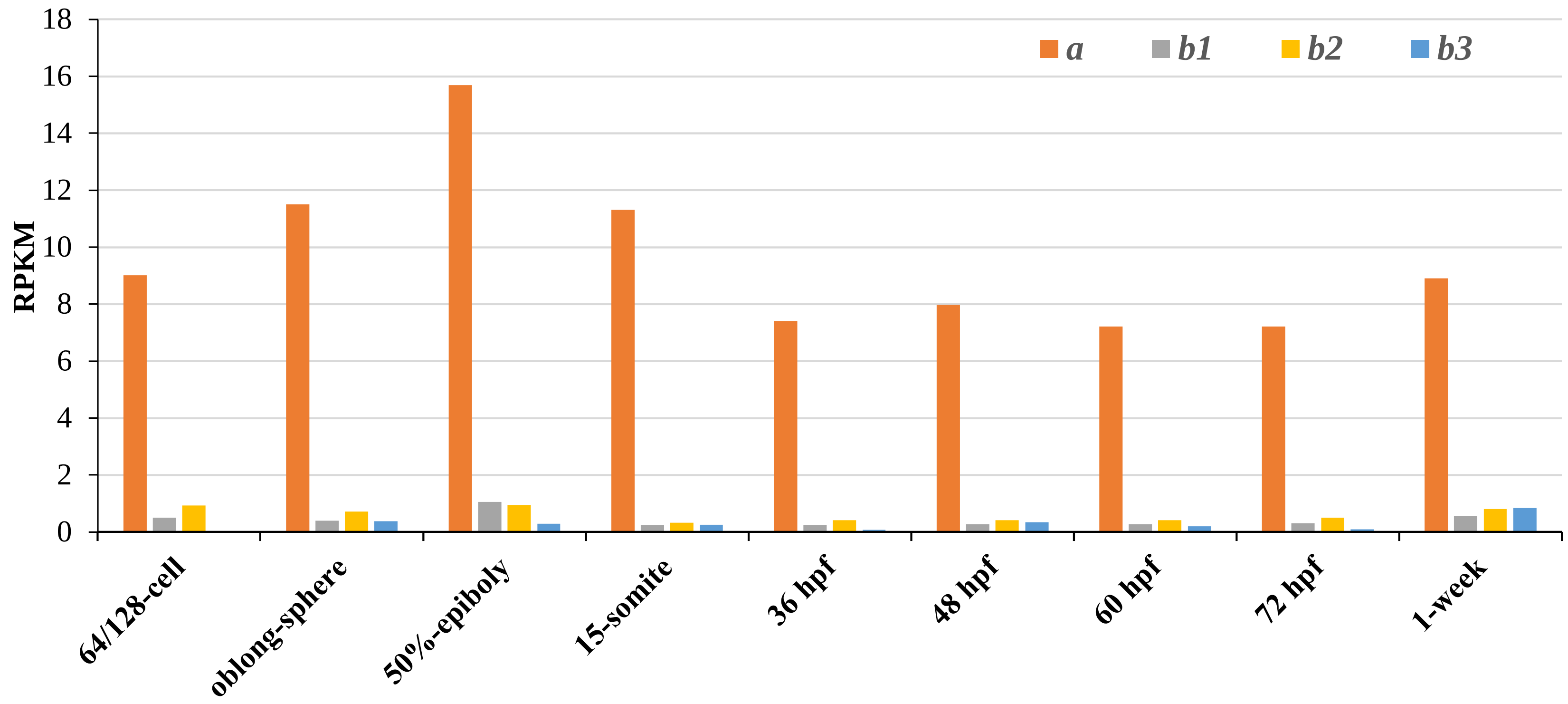

Figure S3. Expression data for *c19orf12* genes during zebrafish development
Expression data for the four genes obtained from a systematic study performed using RNA-Seq on 9 different stages covering 7 major periods (cleavage, blastula, gastrula, segmentation, pharyngula, hatching and early larval stage) in zebrafish development. Expression values are expressed in Reads Per Kilo base per Million mapped reads (RPKM).

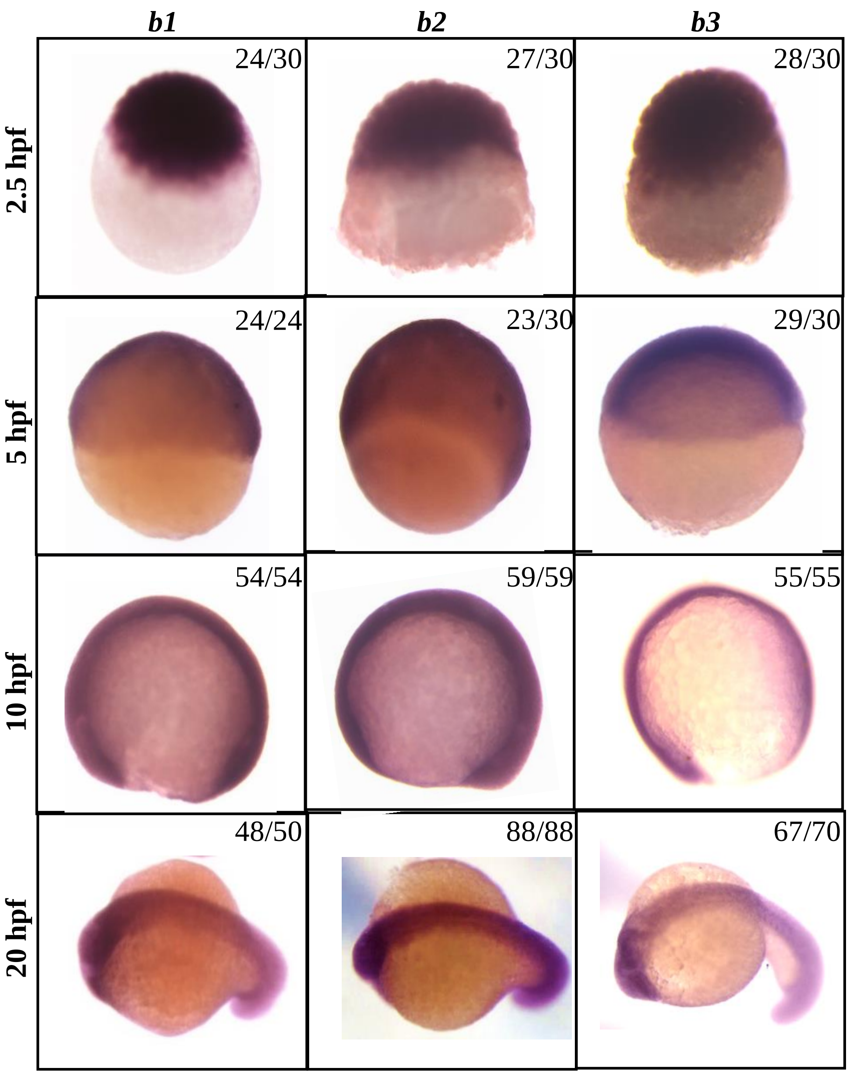

Figure S4. WISH for gene *b1, b2 and b3.*
Representative images of embryos at early stages of development, hybridized with probes specific for gene *b1*, *b2* and *b3*. Numbers in each panel represent embryos used for the experiment and embryos with the result shown in the image. Each hybridization was performed at least twice.

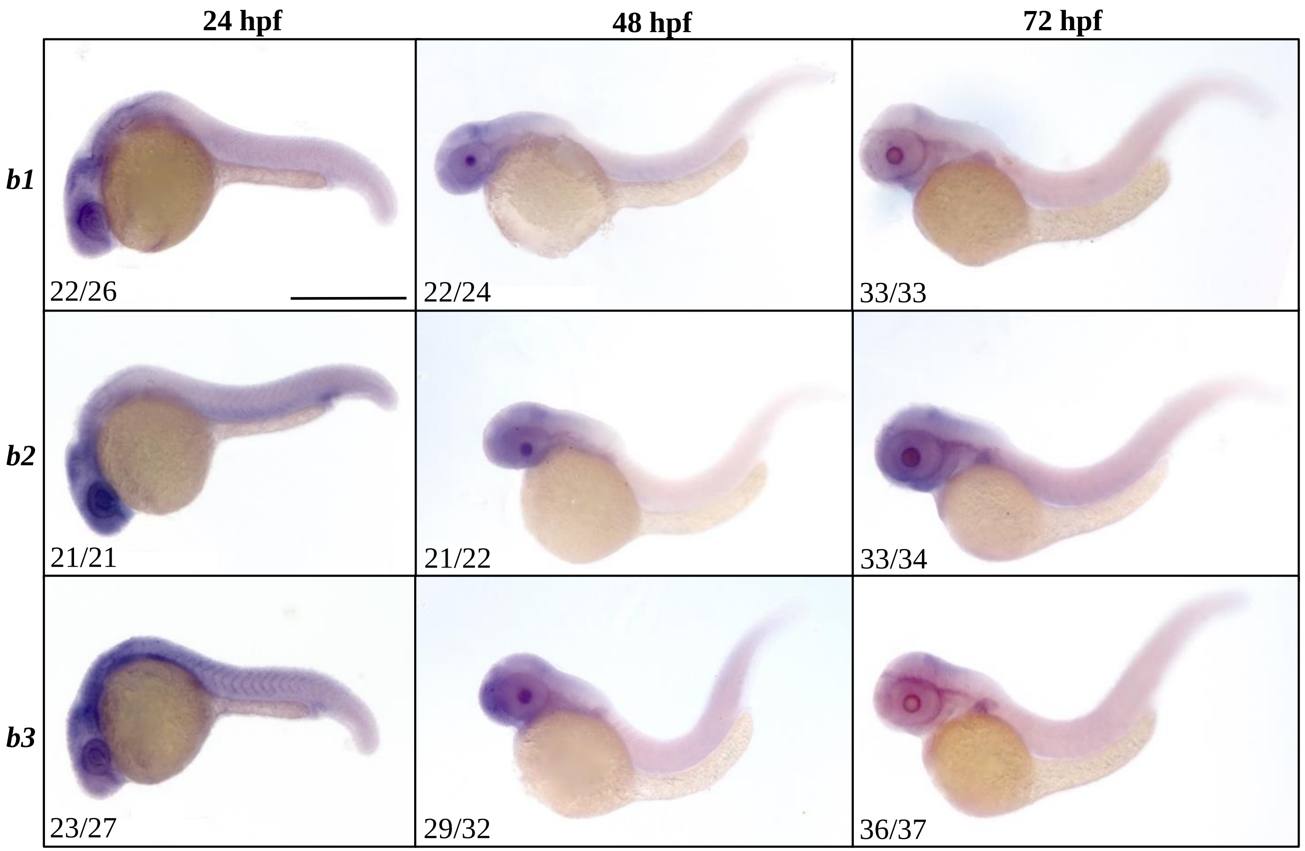

Figure S5. WISH for gene *b1, b2 and b3* at 24, 48 and 72 hpf.
Representative images of embryos at later stages of development, hybridized with probes specific for gene *b1*, *b2* and *b3*. Numbers in each panel represent embryos used for the experiment and embryos with the result shown in the image. Each hybridization was performed at least twice. Scale bar = 500 μm.

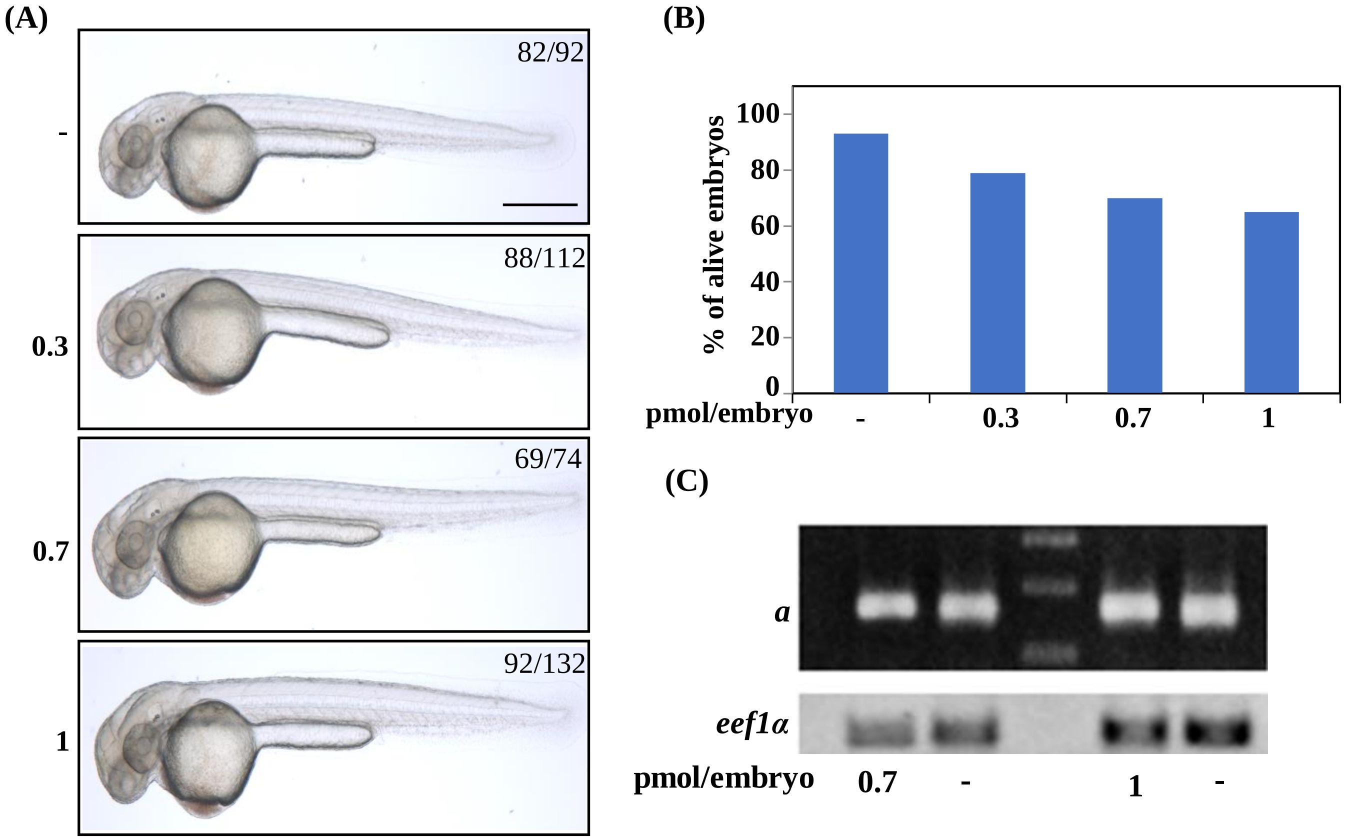

Figure S6. Analysis of the effects induced by the micro-injection of a splice-inhibiting morpholino specific for gene *a*.

(A) Lateral views of embryos (48 hpf) injected with different doses of splice-inhibiting morpholino (SI-MO) specific for gene *a*. (B) Graph showing the percentage of alive embryos (48 hpf) after the injection of the indicated amount of SI-MO. (C) Rapresentative image of the gel electrophoresis of amplification products obtained by RT-PCR of gene *a* and *eef1a* mRNAs in embryos (48 hpf) injected with 0.7 and 1 pmol/embryo of SI-MO.

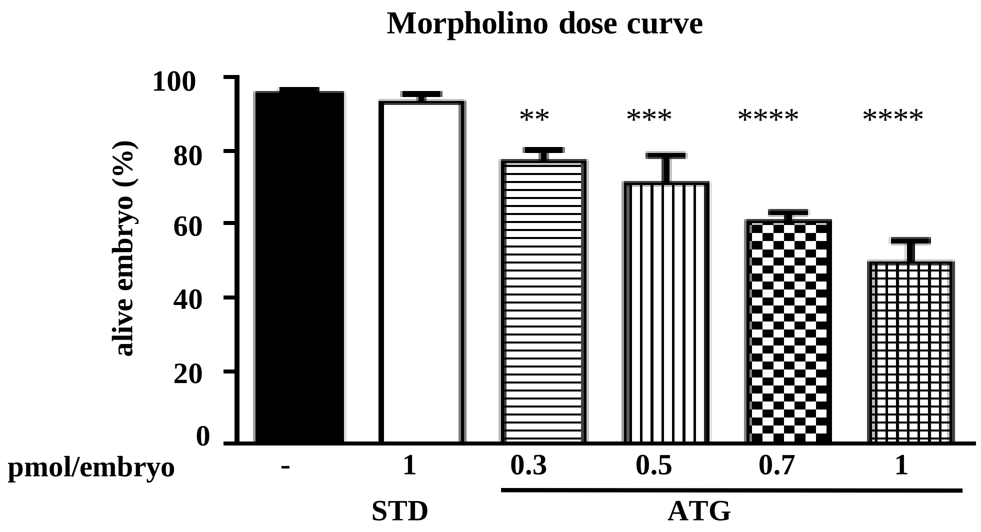

Figure S7. Dose curve of the ATG-blocking morpholino specific for gene *a*.
The graph shows the percentage of alive embryos (48 hpf) after the injection of the indicated amount of control (STD) or gene *a* specific ATG-morpholino (ATG) ** = P<0.01, ***= P<0.001, **** = P<0.0001 (One way ANOVA, with Dunnett's multiple comparisons test)

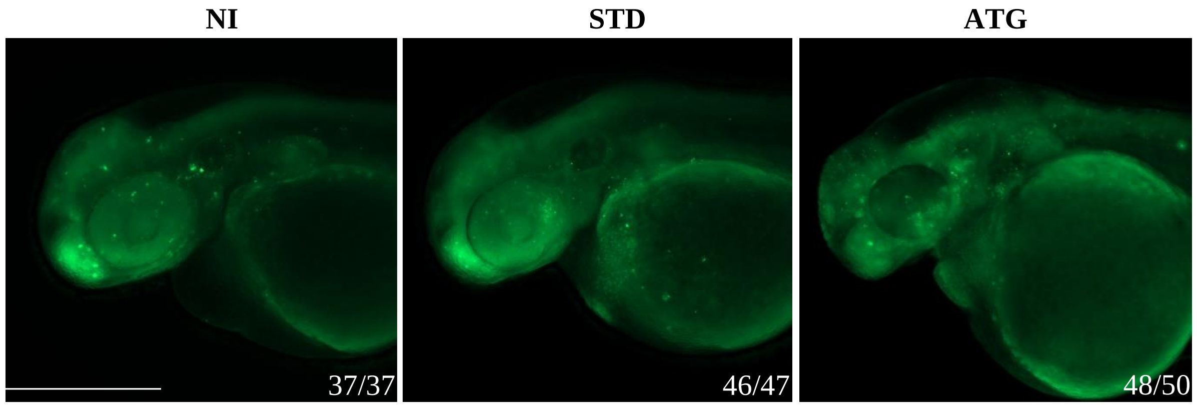

Figure S8. Acridine orange staining.

Rapresentative images of not-injected embryos (NI) or embryos injected with STD and ATG morpholino at 48 hpf, stained with acridine orange, a cell-permeable dye that labels apoptotic cells. Numbers in each panel rapresent the total number of embryos used for the experiment and embryos with the result shown in the image. N = 3. Scale bar = 500 μm

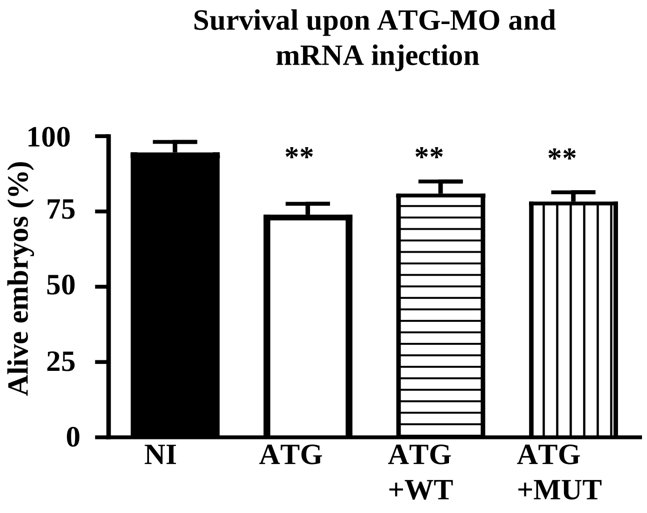

Figure S9. Survival of embryos upon co-injection of human *C19orf12* mRNA with ATG morpholino.

The injection of 150 pmol/embryo of human *C19orf12* mRNA, either wild-type or mutant, together with ATG-blocking morpholino does not modify the percentage of dead embryos at 48 hpf.

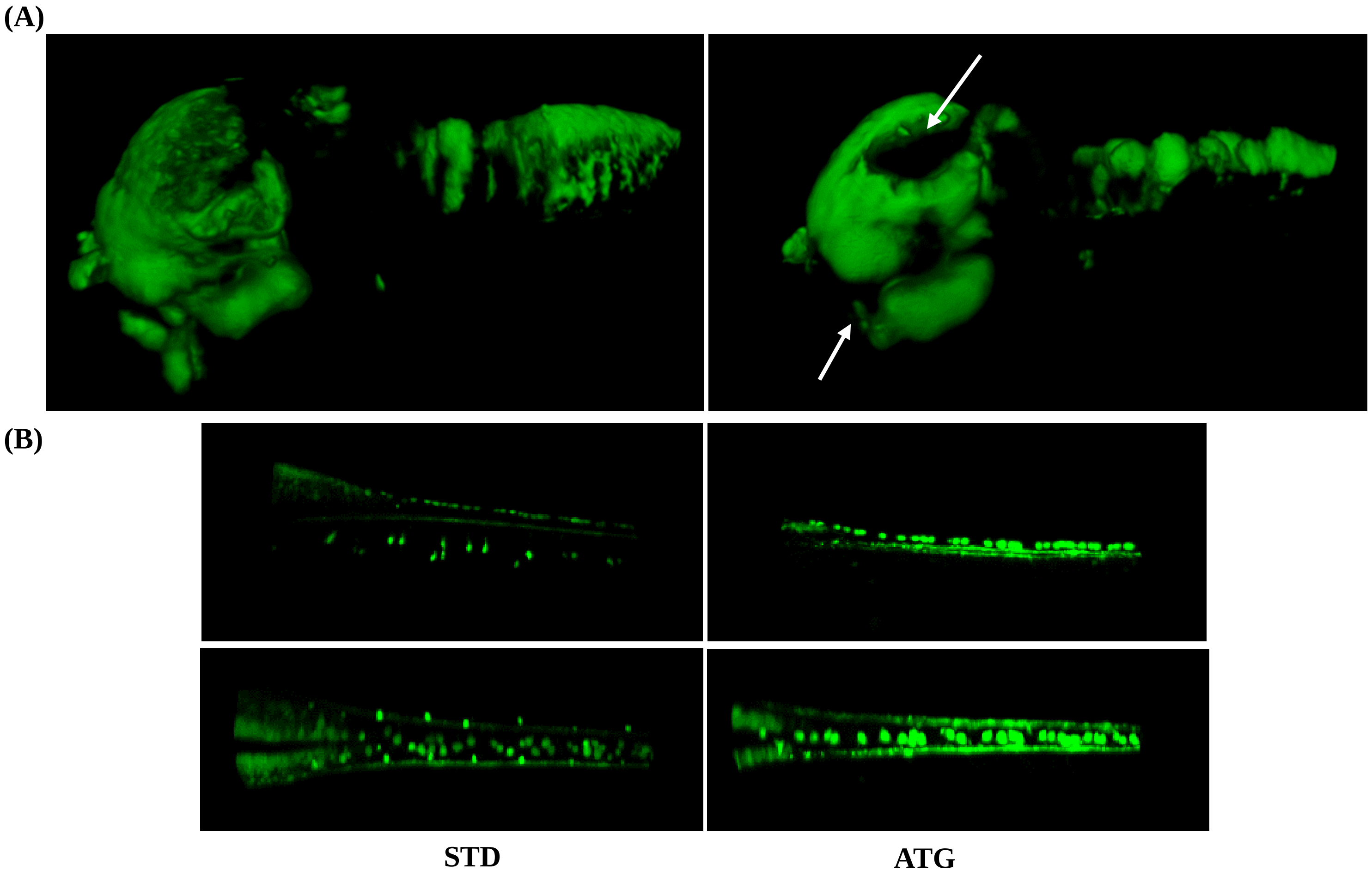

Figure S10. Lightsheet fluorescence analysis of Tg(*neurog1*:EGFP) embryos injected with either STD or ATG morpholino.

(A) Representative lateral views of the rostral part of the embryos. Arrows point to the regions with a clear reduction in fluorescence. (B) Representative lateral and dorsal views of a section of the trunk. Fluorescence in drg neurons is missing in ATG-morphants whereas it appears to be increasd at the level of Rohon Beard neurons. A total of 4 randomly selected embryos were analysed in two biological replicates.

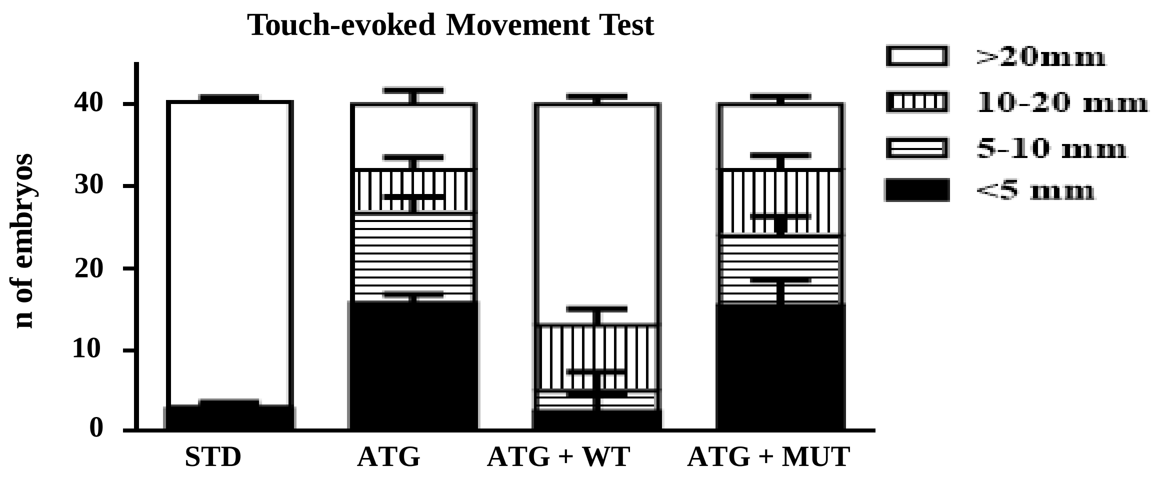

Figure S11. Analysis of the locomotor behaviour by the touch-evoked test.
The graph resumes the results from the analysis of the swimming perfomance of embryos, injected with STD or ATG morpholino, together with either WT or MUT human *c19orf12* mRNA at 48 hpf. Bars represent the percentage of embryos covering distances indicated in the legend.

Sequences of primers	
**Primers**	**Sequence**
**B1 Forward**	**CTTCTGACATGACAATGAGCAG**
**B1 Reverse**	**ACTCTTTATTGGTCACTTTCCATAC**
**B2 Forward**	**TCAAATGGCGAATCGAAAA**
**B2 Reverse**	**CTCTAGTGGTCAATCCTCAAACTT**
**B3 Forward**	**CACAACATAATGAACCGTCAAATA**
**B3 Reverse**	**ACCCACTAATTTTGTCCATATTCT**
**A Forward**	**GATTCATTCGATCGAAATTGC**
**A Reverse**	**CTCACAATAGTGGCGAATCA**
**Sequences of morpholinos**	
**ATG-MO**	**CGGCATAGTGCTTGGAAAGATTACA**
**SI-MO**	**GAAGGACATGAATGTACTTGCCCAC**
**STD-MO**	**CCTCTTACCTCAGTTACAATTTATA**
**p53-MO**	**GCGCCATTGCTTTGCAAGAATTG**

Table S1. Sequences of primers for probe synthesis and morpholinos applied in the study.

ZFIN Gene Name	Abbreviation in the manuscript	Chromosome	Ensembl (GRCz11)
zgc:112052	*(c19orf12)a*	18	ENSDARG00000058857
si:ch211_260e23.7	*(c19orf12)b1*	7	ENSDARG00000104856
zgc:101715	*(c19orf12)b2*	7	ENSDARG00000102929
si:ch211_260e23.8	*(c19orf12)b3*	7	ENSDARG00000105232

Table S2. Nomenclature of *c19orf12* genes in zebrafish

	Hsa-C19orf12	Dre-a	Dre-b1	Dre-b2	Dre-b3
**Hsa-C19orf12**	100.00	59.57	48.23	51.06	55.94
**Dre-a**	59.57	100.00	54.61	60.99	61.70
**Dre-b1**	48.23	54.61	100.00	51.77	58.87
**Dre-b2**	51.06	60.99	51.77	100.00	56.74
**Dre-b3**	55.94	61.70	58.87	56.74	100.00

Table S3 Percentage of identity between human and zebrafish C19orf12 protein sequences.
